# 

**Published:** 1901-05

**Authors:** 


### BOOKS RECEIVED.

Transactions of the American Dermatological Association. Twenty-fourth annual meeting, held at Washington, D. C., May 1 to 3, 1900, in connection with the Fifth Triennial Congress of American Physicians and Surgeons. Edited by Frank Hugh Montgomery, M. D., Secretary. Chicago: P. F. Pettibone & Co. 1901.

The Medical News Pocket Formulary. New (third) edition. Containing 1700 prescriptions representing the latest and most approved methods of administering remedial agents. By E. Quin Thornton, M. D., Demonstrator of Therapeutics, Pharmacy and Materia Medica in the Jefferson Medical College, Philadelphia. Carefully revised to date of issue. In one wallet-shaped volume, strongly bound in leather, with pocket and pencil. Philadelphia and New York: Lea Brothers & Co. 1901. [Price, $1.50 net.

Quiz Compends. No. 4. A compend of human physiology. Especially adapted for the use of medical students By Albert P. Brubaker, A. M., M. D., Adjunct Professor of Physiology and Hygiene in the Jefferson Medical College; Professor of Physiology in the Pennsylvania College of Dental Surgery, etc. Tenth edition, revised and enlarged. Philadelphia: P. Blakiston's Son & Co. 1901. [Price, 80 cents.]

Introduction to the Differential Diagnosis of the Separate Forms of Gallstone Disease; based upon his own experience gained in 433 laparatomies for gallstone. By Professor Hans Kehr, Helbersadt. Authorized translation by William Wotkyns Seymour, A. B. (Yale), M. D. (Harvard); formerly Professor of Gynecology in the University of Vermont; Fellow of the American Association of Obstetricians and Gynecologists; Surgeon to the Samaritan Hospital, Troy, N. Y. Octavo, pages 359. Philadelphia: P. Blakiston's Son & Co. 1901. [Price, $2.50.]

Principles of Surgery. By N. Senn, M. D , Ph. D., LL. D., Professor of Surgery in Rush Medical College in Affiliation with the University of Chicago; Professorial Lecturer on Military Surgery in the University of Chicago; Attending Surgeon to the Presbyterian Hospital; Surgeon-in-Chief to St. Joseph's Hospital; Surgeon-General of Illinois. Third edition, thoroughly revised, with 230 wood-engravings, half-tones and colored illustrations Royal octavo, pages, xiv.—700. Philadelphia; F. A. Davis Co. 1901. [Extra cloth, $4.50 net; sheep or half-russia, $5.50 net.]

A Handbook of Materia Medica, Pharmacy and Therapeutics. Including the physiological action of drugs, the special therapeutics of disease, official and practical pharmacy and minute directions for prescription writing. By Samuel O. L. Potter, A. M., M. D., M. R. C. P., Lond.; formerly Professor of the Principles and Practice of Medicine in the Cooper Medical College of San Francisco; Author of the "Quiz Compends of Anatomy and Materia Medica," etc.; late Major and Brigade-Surgeon of Volunteers, U. S. Army. Octavo, pp. 950. Eighth edition, revised and enlarged. Philadelphia: P. Blakiston's Son & Co. 1901. [Price, $5.00.]

A Text-Book of Gynecology. Edited by Charles A. L. Reed, A. M., M. D., President of the American Medical Association (1900-1901); Gynecologist and Clinical Lecturer on Surgical Diseases of Women at the Cincinnati Hospital; Fellow of the American Association of Obstetricians and Gynecologists; Fellow of the British Gynecological Society, etc. Octavo, pp. 925. Illustrated by R. J. Hopkins. New York: D. Appleton & Co. 1901. [Price, $5.00]

Chronic Urethritis of Gonococcic Origin. By J. De Keersmaecker, Chief of Service, Diseases of the Urinary Organs at the Centralklinik of Antwerp; and J. Verhoogen, Agrégé at the University of Brussels; Chief of Service, Diseases of the Urinary Organs at the Polyclinique Libre. Translated and edited with notes by Ludwig Weiss, M. D., Attending Physician to the Genito Urinary and Skin Service, German Poliklinik; Dermatologist to the Hebrew Orphan Asylum, New York, etc. Small octavo, pp. 274. New York: William Wood & Co. 1901.

International Clinics. A Quarterly of Clinical Lectures and Especially Prepared Articles on Medicine, Neurology, Surgery, Therapeutics, Obstetrics, Pediatrics, Pathology, Dermatology, Diseases of the Eye, Ear, Nose and Throat, and other Topics of Interest to Students and Practitioners, by leading members of the Medical Profession throughout the world. Edited by Henry W. Cattell, A. M., M. D., Philadelphia, with the collaboration of John B. Murphy, M. D., of Chicago; Alexander D. Blackader, M. D., of Montreal; H. C. Wood, M. D., of Philadelphia; T. M. Rotch, M. D., of Boston; E Landolt, M. D., of Paris; Thomas G. Morton, M. D., and Charles H. Reed, M. D., of Philadelphia; J. W. Ballantine, M. D, of Edinburgh, and John Harold, M. D. of London, with regular correspondents in Montreal, London, Paris, Leipsic and Vienna. Volume I. Eleventh series. 1901 Philadelphia: J. B. Lippincott Co. 1901.

Lectures on Nasal Obstruction. By A. Marmaduke Shield, M. B. (Camb.,) F. R. C. S. (Engl.) Surgeon to St. George's Hospital, London, and Surgeon in charge of the Throat Department, etc. 12mo. illustrated. Philadelphia: P. Blakiston's Son & Co. 1901. [Price, $1.50.]

The Feeding of Infants. Home Guide for Modifying Milk. By Joseph E. Winters, M. D., Professor of Diseases of Children, Cornell University Medical College. New York: E. P. Dutton & Co. 1901. [Price, 50 cents]

Memoranda of Poisons. By Thomas Hawkes Tanner, M. D., F.L.S. Eighth revised edition, by Henry Leffmann, A. M., M. D., Professor of Chemistry in the Woman's Medical College of Pennsylvania, etc. Philadelphia: P. Blakiston's Son & Co. 1901. [Price, 75 cents.]

